# The Origin of Hospitals

**Published:** 1887-08-20

**Authors:** 


### The Origin of Hospitals.

The following are a few more contributions to the history of the institution of hospitals, which we select from replies sent in to competition No. 18, Vol I :—

"Hospital," from Latin *hospitium*, French *hôpital*, meant originally simply a place of shelter and entertainment for strangers. The Knights Hospitallers, or Knights of St. John of Jerusalem, founded a hospice at Jerusalem in the middle of the eleventh century for receiving pilgrims from Europe who visited the Holy Sepulchre. This order of Hospitallers afterwards came to England, and settled in the east of London, and founded the Priory of St. John's, Clerkenwell. In 1324, when the Order of the Templars was dissolved, their lands and possessions were given by law to the Brethren of St. John of Jerusalem, "to be employed by them in relieving the poor in hospitalities, celebrating divine worship, and defending the Holy Land." The most ancient English hospital is St. Bartholomew's, founded in connection with a priory of that name by Rahere, minstrel of Henry I, about the year 1122, for the relief of poor sick persons. In 1546 this hospital was re-founded, and given over by the King (Henry VIII) to the City of London, for the reception of a hundred poor and sick persons. St. Thomas's Hospital was originally a religious establishment, founded by Richard, Prior of Bermondsey, in 1213. In the middle of the sixteenth century it became, by purchase, the property of the City of London. In the early years of the eighteenth century Guy's Hospital was founded. Many charitable institutions in England are called hospitals, but the name has been long more generally restricted to places for the reception of the sick and wounded.—K. M.

---

The term *hospitium* originally signified a place of temporary refuge or of entertainment. It gradually developed into that of more permanent reception of the poor and sick, as a work of benevolence, and in course of time from the general sprang the special hospital. We see the original idea in the places of refuge prepared fer pilgrims to Jerusalem, by those who dwelt along their line of route. The latter were glad to give shelter and refreshment to these enthusiastic wanderers, whose example they were unable or unwilling to follow, only craving their prayers at the holy places as a return for their hospitality. In the eleventh century the Latin Church encouraged those who showed faith and penitence bj these pilgrimages, and those who aided them by the way. Christians came out from Jerusalem and escorted these weary ones back into hospital. Within the holy walls was the one in which women were cared for by their own sex only. Pilgrim merchants of Amalfi, Venice, Genoa, celebrated their institution as Hospitallers by founding a house of refuge for pilgrims, A.D. 1046, by the gift of a hospital for the sick in Jerusalem, by favour of royal Baldwin, in 1104. This order assumed later the title of Knights of St. John; but in England, five centuries earlier, St. Austin turned the idol temple given to him by Ethelbert of Kent into the earliest English monastery on record. Hospital, church, and school found a common home in these pious foundations. To all comers the monks were healers, priests, teachers. Under Alfred the Great they practised the healing arts before medicine and surgery were professions. Want of space forbids us to linger over the interesting Diary of Henry II; his charter to Herbaldown Hospital, near Canterbury, given from his sick-bed at Westminster in July 1174, two days after his penance at Becket's tomb; another to St. Leonard's Hospital at York, burnt down in June 1137. But it would be well to observe here that hospitals, so called, were not at this time exclusively parts of monastic organisation, but existed also in their own simplicity, as these charters are quoted by a distinguished and exact authority as given to leper-houses, monasteries, abbeys, and hospitals. It is worthy of observation that oue Ralph de Hospital played a prominent part as Commissioner in a Court of Inquiry held at Windsor by Henry in person. Endless were the useful foundations encouraged by this benevolent and ubiquitous monarch, of which many were suppressed under Henry V. A larger number still under Henry VIII. But continuity may be traced in St. Bartholomew's Hospital, surviving its dissolution as a monastery in 1546, by favour of the King; while the foundation of the Bethlehem Hospital for Lunatics by him is suggestive of specialism. The healing art showed advance by "Clinical Lectures combining practice with theory of medicine," commenced about 1500, the founding of Royal Colleges of Physicians of England, Dublin, and Edinburgh in 1518, 1667, and 1681 respectively. These are landmarks of the progress of hospitals; and in the present day we may say that these houses of refuge afford such tender and careful *hospitium* for the sick and afflicted, that they pass, if God so wills it, from one home to another in peace, not as the Latin orator of old expressed himself: "*Ex vitâ ita discedo, tanquam ex hospitio non tanquam ex domo.*"—A. M. E.

---

The English word "hospital" (often reduced to "spital"), comes from old French *hospital*, now *hôpital*, of which Littré says it was remade from the Latin many centuries ago, although *hospitalis* had given rise quite regularly to "hostel," now "hotel"; the Latin *hospitalis* from *hospes*, a host or guest. The place, however, in which a guest was received was called *hospitium* (Fr. *hospice*); but in course of time the adjective became a noun, and *hospitalis* was adopted in the same sense as *hospitium*, by dropping the noun *domus*. Vitruvius uses *hospitalia* to mean the chambers where guests were received. The origin of our present hospitals is traced in the monastic arrangements for the care of the sick. Every monastery had its *infirmaria*, managed by an *infirmarius*, for the sick and convalescent. In course of time separate buildings were erected for the purpose and special revenues appropriated for their maintenance. The earliest record in England is in the life of Lanfranc, who in 1080 founded two hospitals, one for leprosy and the other for ordinary diseases. The establishments for the sick were in the hands of the clergy till the Reformation, when some of the monasteries and Church property were appropriated and set apart for the use of the sick, as St. Bartholomew's in Smithfield, St. Thomas's in the Borough, Bethlehem and Christ's Hospitals, long known as the five "Royal Hospitals."—Ellice.

